# Factors influencing successful bone union of isolated subtalar arthrodesis for posttraumatic subtalar arthritis: a multicenter case series

**DOI:** 10.1186/s13018-023-04040-9

**Published:** 2023-08-02

**Authors:** Hyong Nyun Kim, Young Rak Choi, Bom Soo Kim, Yu Mi Kim, Jaehyung Lee, Jae Ho Cho, Sunho Cha, Jae Yong Park

**Affiliations:** 1 Department of Orthopaedic Surgery, Kangnam Sacred Heart Hospital, Hallym University College of Medicine, Seoul, Republic of Korea; 2 Department of Orthopaedic Surgery, Asan Medical Center, University of Ulsan College of Medicine, Seoul, Republic of Korea; 3 Department of Orthopaedic Surgery, Inha University Hospital, Incheon, Republic of Korea; 4 Department of Orthopedic Surgery, Sanbon Hospital, Wonkwang University College of Medicine, Gunpo-si, Gyeonggi-do Republic of Korea; 5 Department of Orthopaedic Surgery, Hallym University Sacred Heart Hospital, Hallym University College of Medicine, 22, Gwanpyeong-ro 170 beon-gil, Dongan-gu, Anyang-si, Gyeonggi-do Republic of Korea; 6 Department of Orthopaedic Surgery, Chuncheon Sacred Heart Hospital, Hallym University College of Medicine, Chuncheon-si, Gangwon-do Republic of Korea

## Abstract

**Background:**

The purpose of this study was to find the factors influencing successful bone union for isolated subtalar arthrodesis in posttraumatic subtalar arthritis following calcaneal fracture.

**Material and methods:**

We retrospectively analyzed the rate of successful bone union of 119 cases of isolated subtalar arthrodesis for posttraumatic subtalar arthritis performed at five university hospitals between January 2010 and December 2019. Multivariate logistic regression analysis was used to find the factors associated with successful bone union. Successful bone union was defined as resolution of hindfoot pain with the presence of osseous trabecular bridging involving more than 50% of the posterior facet within 6 months postoperatively.

**Results:**

There were 77 (64.7%) cases of successful bone union, 11 (9.2%) cases of delayed union, 8 (6.7%) cases of questionable union, and 23 (19.3%) cases of nonunion. Use of fully threaded screws was 5.90 times [odds ratio (OR) = 5.90, 95% confidence interval (CI) = 1.42–24.49, *p* = 0.02] more likely to achieve successful bone union compared to the use of partially threaded screws. Use of two parallel screws or the two divergent screws were 3.71 times (OR = 3.71, 95% CI = 1.05–13.14, *p* = 0.04) and 4.65 times (OR = 4.65, 95% CI = 1.23–17.53, *p* = 0.02) more likely to achieve successful bone union compared to the use of a single screw. Use of cancellous autograft or structural autograft was 4.72 times (OR = 4.72, 95% CI = 1.17–19.06, *p* = 0.03) and 7.12 times (OR = 7.12, 95% CI = 1.46–34.68, *p* = 0.02) more likely to achieve successful bone union compared to no graft use.

**Conclusion:**

Use of fully threaded screws, autograft, and two screws compared to a single screw were the factors associated with successful bone union within six postoperative months after subtalar arthrodesis for the posttraumatic arthritis.

**Supplementary Information:**

The online version contains supplementary material available at 10.1186/s13018-023-04040-9.

## Introduction

**Fig. 1:**
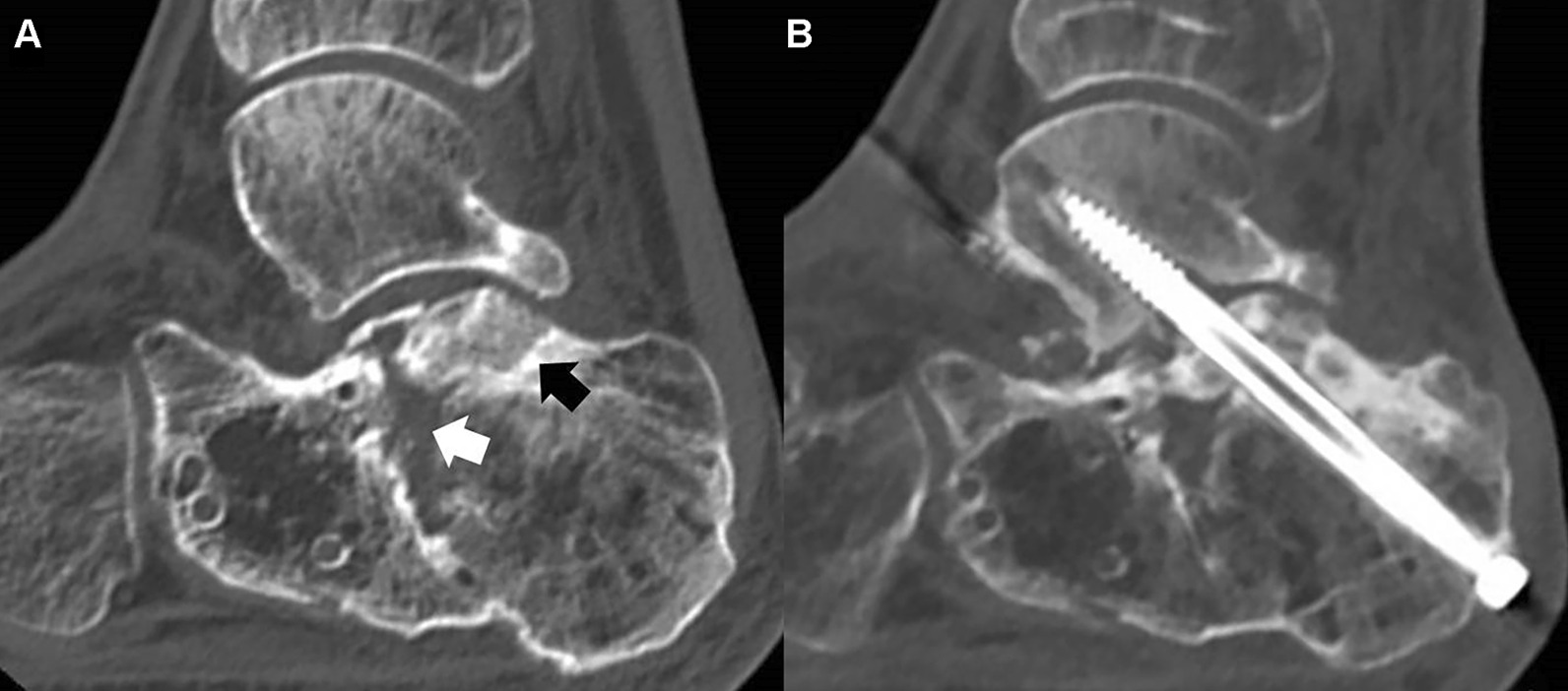
**a** Posttraumatic subtalar arthritis after calcaneal fracture can be different from other etiologies of subtalar arthrodesis because of the potential for insufficient blood supply to the posterior facet fragment. During fracture reduction, the collapsed posterior facet fragment is elevated from the body of the calcaneus, and an empty space is created underneath the fragment (white arrow). Although this empty space can be filled with bone at a later time, the blood supply to the posterior facet fragment can be compromised and can result in bone sclerosis (black arrow). **b** This can negatively affect joint fusion

Subtalar arthrodesis is performed to treat symptomatic subtalar arthritis not responding to conservative treatment or is used to correct congenital and acquired deformities [1–5]. The outcomes of subtalar arthrodesis have generally been reported as favorable, but complications such as nonunion are still encountered [6, 7]. Many papers report that nonunion is not as uncommon as previously thought [2, 4, 7, 8]. In a large cohort study of 184 consecutive isolated subtalar arthrodesis, a 16% rate of nonunion was reported^2^. In that study, nonunion was significantly higher among patients with avascular bone at the site of the arthrodesis, and the presence of avascular bone was usually observed in association with a traumatic etiology. In a study of 115 patients who underwent subtalar arthrodesis for posttraumatic subtalar arthritis, osseous consolidation was considered definite in only 55% of cases and was questionable in 21% of cases, while nonunion was observed in 24% of cases [5]. Posttraumatic subtalar arthritis after calcaneal fracture can be different from other etiologies of subtalar arthrodesis because of the potential for insufficient blood supply or avascular sclerotic bone at the joint [2, 9, 10]. During calcaneal fracture reduction, the collapsed posterior facet fragment is elevated from the body of the calcaneus, and an empty space is created underneath the fragment where the bone graft is known to be not necessary. Although this empty space can be filled with bone at a later time, the blood supply to the posterior facet fragment can be compromised and can result in avascular necrosis of the bone [2]. Insufficient blood supply or avascular sclerotic bone at the joint can negatively affect joint fusion (Fig. 1). Furthermore, in cases where there is depression or step-off at the posterior facet, it may be difficult to compress the calcaneus and talus for full bony contact at the joint to help increase stability. These factors should be considered for successful subtalar arthrodesis for posttraumatic subtalar arthritis. However, there are relatively few studies that have focused on subtalar arthrodesis only for treatment of posttraumatic subtalar arthritis following calcaneal fracture [4, 5, 9, 10].

Whether the reason for the subtalar arthrodesis is posttraumatic or nontraumatic, nonunion is a debilitating result for patients, causing significant pain during walking [4, 5]. Risk factors for nonunion have previously been characterized as: cigarette smoking, diabetes, prior ankle fusion, and revision surgery [1–5]. Preventing nonunion is important and should be the aim of surgery. However, it can sometimes take more than a year to achieve bone consolidation and for the patient to be free of pain and return to activities of daily life and full employment^5^. In such cases, success of treatment remains questionable even where bone union is ultimately achieved [4]. Therefore, rapid and reliable bone healing that allows patients to return to normal activities as quickly as possible should also be the aim of surgery. Although several studies have analyzed risk factors for nonunion after subtalar arthrodesis, few studies focus on the factors for a rapid and reliable bone union after subtalar arthrodesis used specifically for posttraumatic arthritis. We regarded the subtalar arthrodesis to be successful when bone union could be confirmed within six postoperative months and combined with resolution of hindfoot pain. Questionable union, delayed union, or nonunion were deemed unsuccessful surgical outcomes. In this study, we focus on the factors that can managed by surgeons to increase early and definitive union.

## Materials and methods

### Study design

This multicenter study reviewed 119 cases of isolated subtalar arthrodesis for posttraumatic subtalar arthritis following calcaneal fracture performed at five university hospitals between January 2010 and December 2019 by five foot and ankle fellowship-trained orthopedic surgeons. The study was approved by the Institutional Review Boards of each participating hospital and all methods were performed in accordance with the relevant guidelines and regulations. Cases of patients undergoing isolated subtalar arthrodesis for posttraumatic subtalar arthritis following calcaneal fracture were included in the study. Cases of subtalar arthrodesis without a history of calcaneal fracture, or those treated in combination with ankle arthrodesis, ankle arthroplasty, or triple arthrodesis were excluded. Patients with uncontrolled diabetes, Charcot neuroarthropathy or infection around the subtalar joint were also excluded from this study. Medical records were reviewed for basic patient demographic data, including age, sex, cigarette smoking, diabetes, body mass index (BMI) in kg/m^2^, and nonunion after prior subtalar arthrodesis, which are known to be the risk factors for nonunion after isolated subtalar arthrodesis. Patients’ operative records and postoperative radiographs were reviewed for type of screw used (partially, or fully threaded), screw configuration (single [11], parallel [12], divergent [13], or delta [14]), number of screws used, and use of bone graft (no graft, bone substitutes, allograft, cancellous autograft, and structural autograft). Patients’ informed consent was waived from the IRB as the study was on the patients’ medical records and radiographs.

### Operative technique

**Fig. 2:**
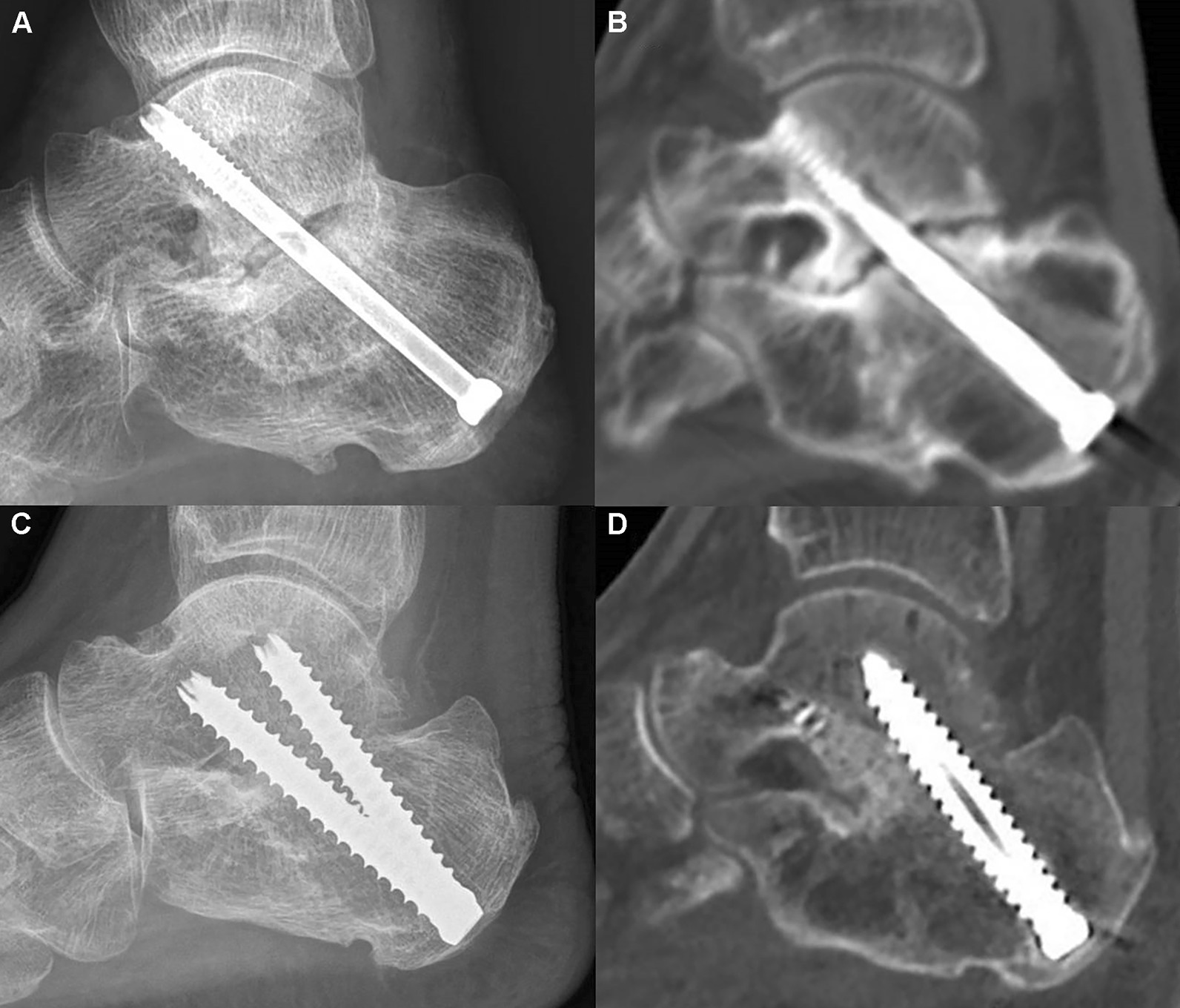
**a**,** b** Nonunion was observed on the lateral radiograph and CT scan of a 67-year-old male patient after using a single partially threaded screws for the subtalar arthrodesis. **c**, **d** Bone union was observed in a 56-year-old male patient after using two fully threaded screws in divergent configuration

Subtalar arthrodesis was performed by different surgeons utilizing a common technique; denudement of cartilage and subchondral bone until bone bleeding was seen. Screws used were either fully threaded 6.5 or 7.0 mm cannulated screws or partially threaded cannulated screws (Fig. 2). One or two screws were inserted into the calcaneal tuberosity and through the talus or with an additional screw inserted from the lateral calcaneus to form a delta configuration.

**Fig. 3:**
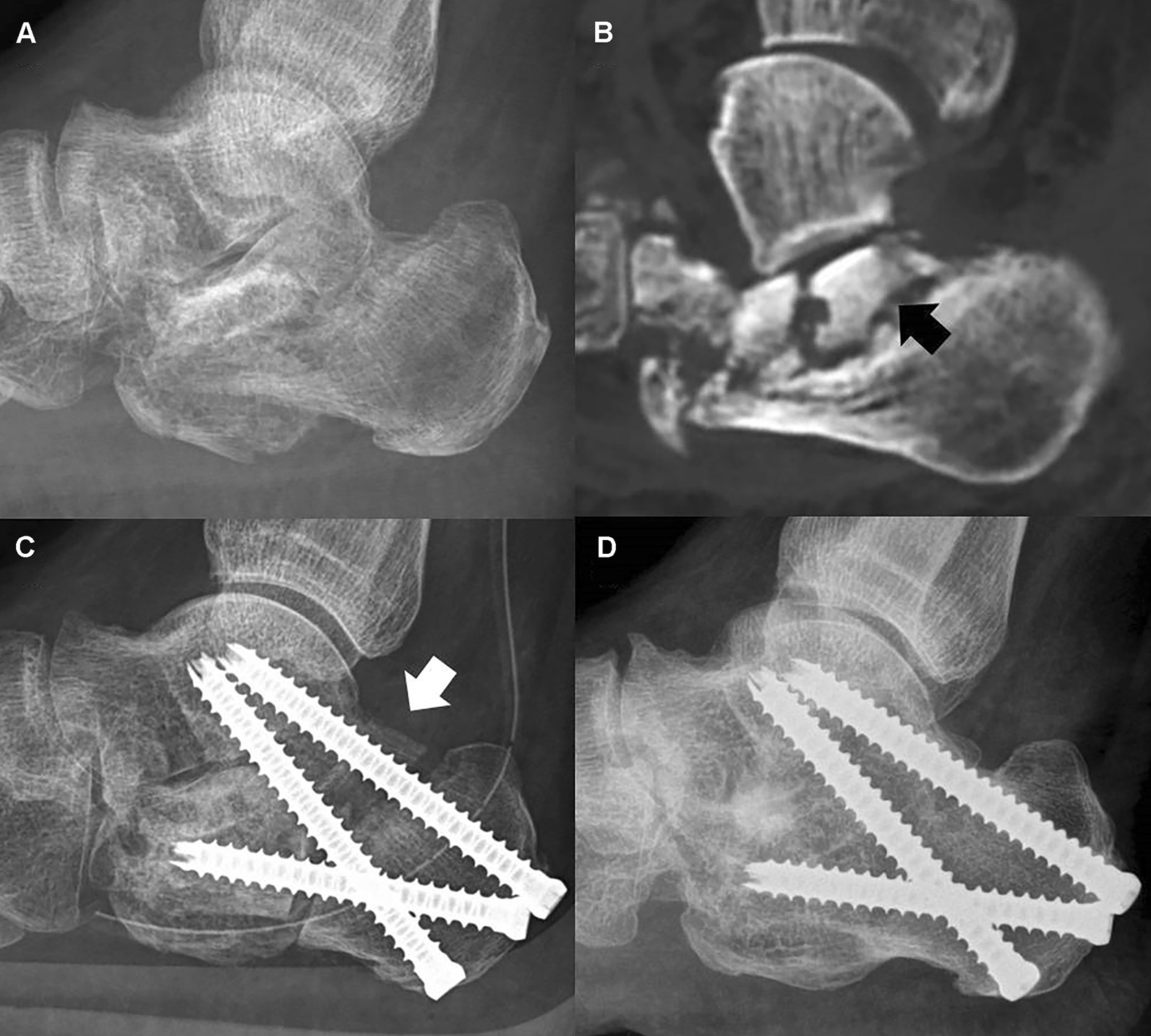
Avascular bone (black arrow) at the posterior facet of the calcaneus was observed on the **a** plain radiograph and **b** CT scan image after the pin fixation of a calcaneal fracture. **c** A large talocalcaneal gap left behind after removal of the avascular bone was filled with autoiliac tricortical bone (white arrow) which was fixed with two fully threaded screws. **d** Osseous consolidation was confirmed on the plain radiograph

Autograft from the iliac crest, local autograft from the lateral calcaneal wall exostectomy, allograft, or bone substitute was inserted to fill any appreciable subtalar gap. There was not a uniform criterion for bone grafting. The use of bone grafting or numbers and types of screw depended on surgeons’ preference rather than the cases. In cases of sclerotic avascular bone of the posterior facet, avascular bone was removed and any gap between talus and calcaneus of more than 1 cm was filled with autoiliac tricortical bone graft and fixed with one or two fully threaded screws (Fig. 3). Postoperatively, the hindfoot was immobilized for 4–6 weeks and weightbearing was allowed after 8–12 weeks from the time of surgical fixation.

### Outcome assessments

The primary outcome measure was osseous consolidation at the subtalar joint. Bone union was evaluated by lateral foot radiograph with both-oblique and hindfoot axial images or computed tomography (CT) scan and based upon clinical features, such as persistence of pain or functional disabilities. Where simple radiographic and clinical evaluation remained inconclusive, CT scan evaluation was used to determine the extent of osseous consolidation [3]. Successful bone union was defined as resolution of hindfoot pain with the presence of osseous trabecular bridging of more than 50% of the posterior facet surface without evidence for screw loosening before the 6-month postoperative stage [15, 16]. Delayed union was defined as bridging callus or trabeculation observed beyond 6 months from the surgery. Nonunion was defined as the lack of bridging callus or trabeculation and continued pain for more than 1 year postoperatively, or any case requiring revision arthrodesis. Where some degree of bridging callus or trabeculation was observed but associated clinically with significant pain, the case was classified as questionable union. Screw loosening was defined as any discernible radiolucent halo around the screw or when the screw had moved from its immediate postoperative position. Radiologic union was independently assessed by musculoskeletal radiologists. Outcomes were deemed successful for cases that achieved resolution of hindfoot pain combined with evidence of adequate bone union within six postoperative months.

### Statistical analysis

To estimate the association between variable factors such as age, sex, BMI, cigarette smoking, diabetes, revision subtalar arthrodesis, type of screw used (partially, or fully threaded), number of screws used (single, two- and multiple-screw techniques), type of configuration (single, parallel, divergent, and delta configuration), use of bone graft (no graft, bone substitutes, allograft, cancellous autograft, and structural autograft) and the binary outcomes (successful bone union or failure of successful bone union), each predictor variable was first analyzed individually calculating odds ratios (OR) and their 95% confidence intervals (CI) in bivariate analysis. We then fitted the full multivariate logistic regression model including all potential predictors. Predictors were excluded if the p value of the log likelihood ratio test was greater than 0.10. Predictor exclusion was continued until all remaining predictors had *p* < 0.10. Only five of 119 cases used a delta screw configuration, and these cases were excluded from the multivariate regression analysis. In the nonunion outcome model, given the large number of predictor variables with a relatively low number of events (23 nonunions), an interactive forward variable selection was done using explanatory values with *p* < 0.05, to help mitigate potential overfitting. Statistical analyses were performed using the SPSS version 21.0 (IBM Corporation, Armonk, NY, USA). Differences with p values of less than 0.05 were considered statistically significant.

## Results

The results of our review are summarized in Table 1. Successful bone union with osseous consolidation and resolution of hindfoot pain within six postoperative months was achieved in 77 (64.7%) cases, while 11 (9.2%) cases resulted in delayed union, eight (6.7%) cases resulted in a questionable union, and 23 (19.3%) cases were deemed to reflect that of a bony nonunion.

**Table 1:** Successful bone union rates according to variable factors

	Number of cases @N = 114^a^	Successful bone union (union rate)^b^	Collinearity statistics		Bivariate analysis: chi-square test		Multivariate analysis: binary logistic regression	
			Tolerance	VIF	Odds ratio (95% CI)	*p* value^c^	Odds ratio (95% CI)	*p* value^d^
Male	83 (72.8%)	50 (60.2%)	0.91	1.10	0.62 (0.25–1.51)	0.29		
Old age (> 65 years)	12 (10.5%)	7 (58.3%)	0.77	1.29	0.80 (0.35–3.48)	0.71		
Overweight (BMI > 25 kg/m^2)^	47 (41.2%)	30 (61.2%)	0.95	1.05	0.77 (0.24–2.70)	0.51		
Diabetes	16 (14.0%)	5 (31.3%)	0.75	1.33	0.21 (0.07–0.66)	0.004	0.13 (0.03–0.61)	0.01
Smoking	25 (21.9%)	16 (64.0%)	0.87	1.15	1.05 (0.42–2.64)	0.92		
Revision subtalar arthrodesis	8 (7.0%)	6 (75.0%)	0.92	1.10	1.81 (0.35–9.45)	0.47		
Type of screw			0.74	1.35				
Partially threaded screw	78 (68.4%)	39 (50.0%)			Reference	–	Reference	–
Fully threaded screw	36 (31.6%)	33 (91.7%)			11.00 (3.11–38.88)	< 0.001	5.90 (1.42–24.49)	0.02
Type of configuration			0.79	1.27				
Single (1 screw)	29 (25.4%)	10 (34.5%)			Reference	–	Reference	–
Parallel (2 screws)	33 (28.9%)	23 (69.7%)			4.37 (1.50–12.70)	0.01	3.71 (1.05–13.14)	0.04
Divergent (2 screws)	52 (45.6%)	39 (75.0%)			5.70 (2.12–15.34)	< 0.001	4.65 (1.23–17.53)	0.02
Bone graft			0.86	1.17				
No bone graft	29 (25.4%)	11 (37.9%)			Reference	–	Reference	–
Bone substitute	12 (10.5%)	6 (50%)			1.64 (0.42–6.36)	0.48	1.05 (0.21–5.19)	0.95
Allograft	20 (17.5%)	11 (55.0%)			2.00 (0.63–6.36)	0.24	2.57 (0.63–10.53)	0.19
Cancellous autograft	25 (21.9%)	19 (76.0%)			5.18 (1.58–16.95)	0.004	4.72 (1.17–19.06)	0.03
Structural autograft	28 (24.6%)	25 (89.3%)			13.6 (3.32–56.03)	< 0.001	7.12 (1.46–34.68)	0.02

*VIF* Variance influence factor, *CI* Confidence interval, *Ref* Reference

^a^Values are given as the number of cases with percentages in parenthesis

^b^Successful bone union was defined as resolution of hindfoot pain with the presence of osseous trabecular bridging of more than 50% of the posterior facet surface without evidence for screw loosening before the 6-month postoperative stage. Values are given as the number of cases with the successful bone union and the union rate in parenthesis

^c^Chi-square test or Fishers’ exact test was used to determine the association between the variable factors and the successful bone union

^d^Multivariate logistic regression analysis was performed to determine the association between the variable factors and the successful bone union

There were 5 cases with the screw penetrating to the ankle joint, 18 cases with screw loosening that led to nonunion and 9 cases with screw loosening that eventually achieved bone union. Use of fully threaded screws, two parallel or two divergent screws, and use of autograft, were the variables associated with successful bone union within six postoperative months after subtalar arthrodesis for posttraumatic subtalar arthritis based on multivariate logistic regression analysis. The Hosmer–Lemeshow goodness of fit test (*X*^2^ = 4.94, *p* = 0.76) showed that the model had a good calibration degree. The presence of diabetes decreased the probability of successful bone union (OR = 0.13, 95% CI = 0.03–0.61, *p* = 0.01). Use of fully threaded screws was 5.90 times (OR = 5.90, 95% CI = 1.42–24.49, *p* = 0.02) more likely to achieve successful bone union compared to using partially threaded screws in the multivariate regression analysis. Use of two parallel screws or the two divergent screws were 3.71 times (OR = 3.71, 95% CI = 1.05–13.14, *p* = 0.04) and 4.65 times (OR = 4.65, 95% CI = 1.23–17.53, *p* = 0.02) more likely to achieve successful bone union compared to the use of a single screw. Use of cancellous autograft or structural autograft was 4.72 times (OR = 4.72, 95% CI = 1.17–19.06, *p* = 0.03) and 7.12 times (OR = 7.12, 95% CI = 1.46–34.68, *p* = 0.02) more likely to achieve successful bone union compared to no graft use.

The union rates of the cases that used fully threaded screws (Additional file 1), two parallel or divergent screws (Additional file 2), and autografts (Additional file 3) were significantly higher compared to that of the cases that used partially threaded screws, a single screw, and no graft. However, the number and the configuration of screws and the use of bone graft were not evenly distributed between the study groups that they were not comparable.

We also studied the factors that were associated with nonunion (Additional file 4). The presence of diabetes increased the probability of nonunion by 5.38 times (OR = 5.38, 95% CI = 1.51–19.18, *p* = 0.01). Use of two screws was 82% (OR = 0.18, 95% CI = 0.06–0.55, *p* = 0.002) less likely to develop nonunion compared to the use of a single screw in the multivariate regression analysis. Furthermore, use of autograft was 79% (OR = 0.21, 95% CI = 0.06–0.76, *p* = 0.02) less likely to develop nonunion compared to use of no graft.

## Discussion

The most important finding of the present study was demonstrating that the use of fully threaded over partially threaded screws, use of two screws compared to use of a single screw, and use of autograft compared to no graft use were the factors associated with successful bone union within six postoperative months of arthrodesis.

**Fig. 4:**
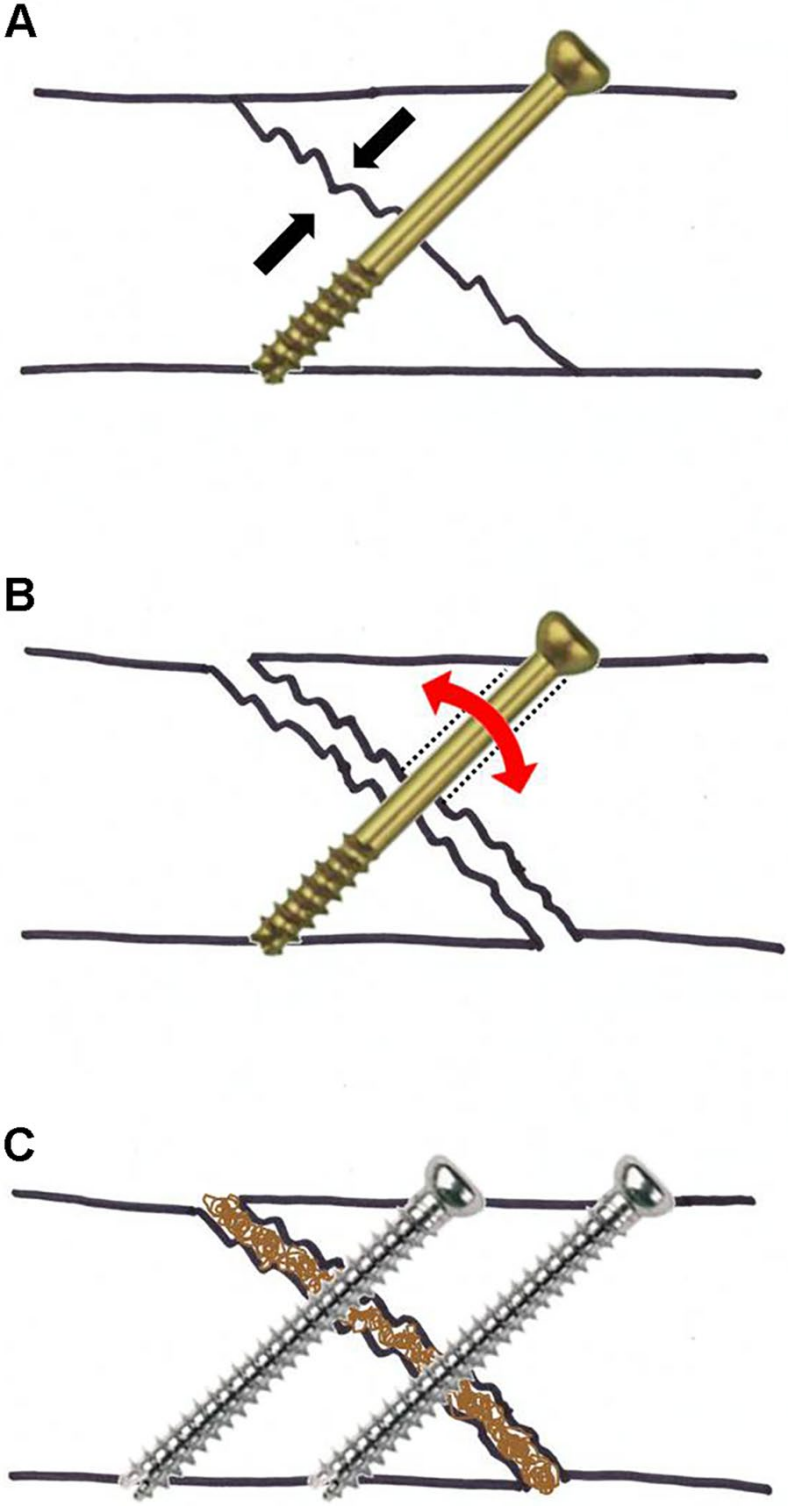
**a** A partially threaded lag screw is effective on the premise that the fracture fragment achieves full contact with the fractured bone. The fracture fragment is fully opposed against the fractured bone and is fixed between the screw head and the frictional force generated at the fracture surface. **b** In a situation where contact between the fractured bones cannot be achieved, the smooth unthreaded shaft of the screw cannot gain purchase to adequately hold the bone together, allowing bone movement (arrow) leading to screw loosening (dotted line) and failure of fixation. **c** Use of fully threaded screws can afford the bony purchase for better opposition and fixation of fractured surfaces. In such cases, bone grafting can enhance bone union

**Fig. 5:**
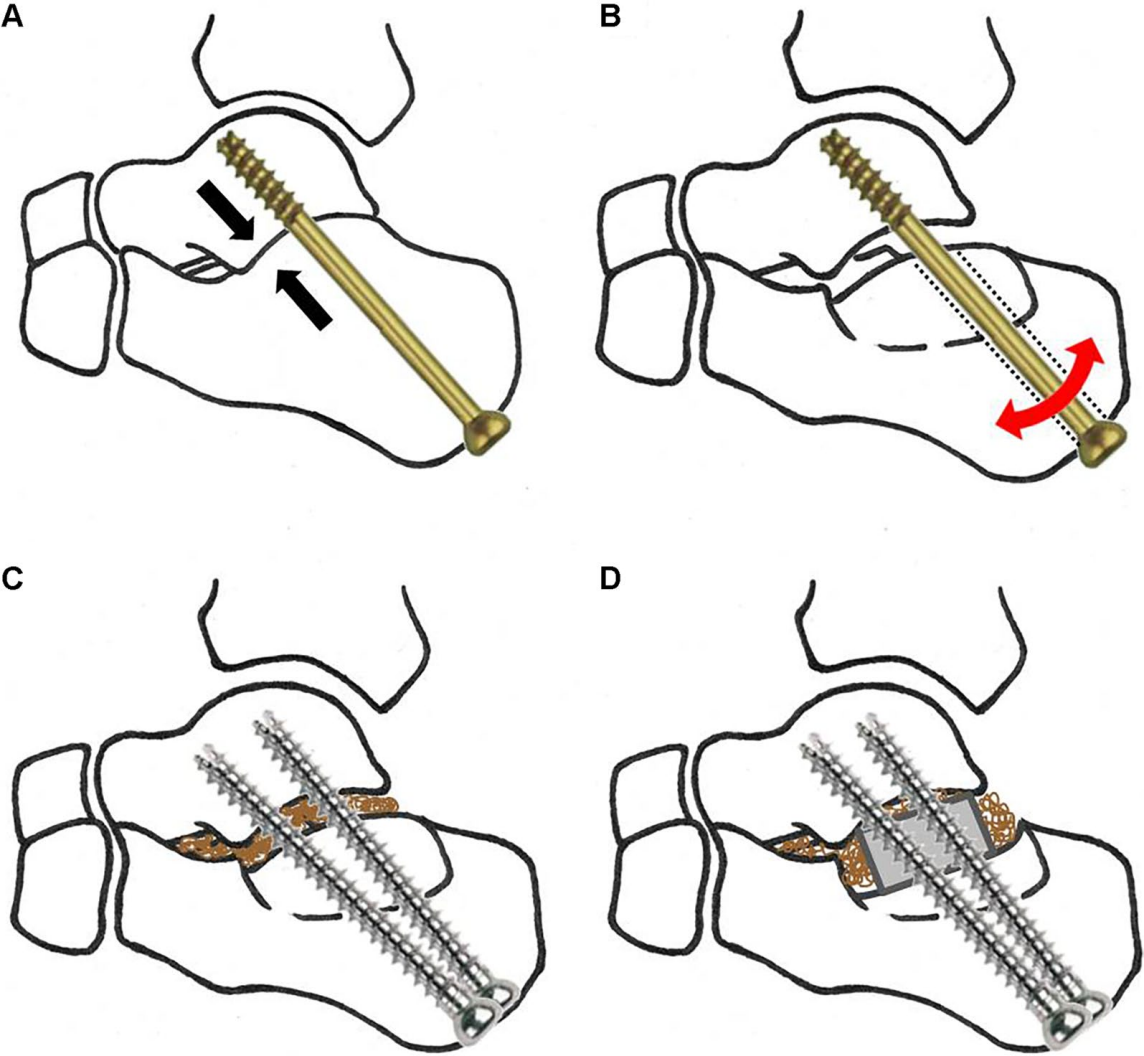
**a** Subtalar arthrodesis is performed using a large diameter partially threaded screw as a lag screw intended to compress the calcaneus with the talus for a stable fixation. **b** In a situation where full contact of talus and calcaneus cannot be achieved, particularly in cases with a depressed calcaneal posterior facet or when a wide gap is created after resection of avascular bone, the smooth unthreaded shaft of the screw may not gain bony purchase enough to hold the calcaneus. **c** In this situation, use of fully threaded screws can help gain purchase to both bones and be a better option for achieving a stable fixation. A bone grafting may enhance bone union. **d** A structural autograft can enhance bone union in cases of a wide gap between talus and calcaneus after resection of avascular bone

Two factors are known to be the key to successful bone union: the mechanical stability of fixation; and the biologic factors that play a pivotal role in osseous consolidation [7, 17]. Most of the cases of subtalar arthrodesis reported in the literature were performed utilizing the Arbeitsgemeinschaft für Osteosynthesefragen (AO) Working Group for Osteosynthesis principles for fracture treatment using large diameter partially threaded screws as a lag screw to compress the calcaneus toward the talus for a stable fixation (Fig. 3) [2, 7, 18–20]. Use of more than one screw and creating a delta configuration are treatment features proven to add stability to subtalar fixation in biomechanical studies [13, 14, 18, 21]. However, subtalar arthrodesis for posttraumatic subtalar arthritis with depression or step-off at the posterior facet can be different from fracture fixation using the lag screw technique, because sometimes it is difficult to achieve full contact between bones at the joint. Where there is a large amount of avascular bone, removal of such sclerotic avascular subchondral bone until there is bone bleeding can result in a wide gap formed between talus and calcaneus. Partially threaded lag screws are effective on the premise that the fracture fragment achieves full contact with the fractured bone (Fig. 4a) [17, 22–24]. However, in a situation where contact between the fractured bones cannot be achieved, the smooth unthreaded-shaft of the screw cannot gain enough purchase to hold the bone, allowing bone movement and leading to screw loosening and to failure of fixation (Fig. 4b) [25]. Where such full contact of bones cannot be achieved or when compression cannot be maintained by a partially threaded lag screw, use of fully threaded screws can be a better option to hold both bones and achieve stable fixation (Fig. 4c) [24, 26]. Further, the subtalar joint sustains shear forces during weightbearing because the subtalar joint axis is inclined about 41 degrees from the horizontal foot axis [14, 27–31]. In a mechanical study of 36 sawbone blocks to test shear strength and resistance, use of fully threaded screws had significantly greater stiffness against shear forces compared to the use of partially threaded lag screws (106.1 vs 80.1 N/mm, *p* = 0.03) [26]. In a biomechanical study on 10 pairs of cadaveric subtalar joints, two fully threaded tapered screws for subtalar joint fixation provided markedly better resistance to rotation than identically placed conventional partially threaded lag screws, with an approximately fourfold increase in torsional stiffness and a corresponding decrease in the amount of joint rotation in response to provoking torsional loads [23]. What drew our attention in other studies was the high union rate after subtalar distraction bone block arthrodesis. A systemic review of subtalar distraction arthrodesis reported a 94% of union rate in 492 total cases from 24 studies [31]. Chahal et al. [15] reported a significantly higher union rate of 95% after a subtalar bone block distraction arthrodesis compared to a 65% rate of bone union after a standard in situ subtalar arthrodesis without bone grafting (*p* < 0.05). Many factors can be attributed to the high rate of bone union achieved from subtalar distraction arthrodesis compared to the standard in situ subtalar arthrodesis. One reason may be that distracting the joint forcibly and inserting a bone block achieves full bone contact and use of fully threaded screws to maintain bone height increases the stability of fixation. On the other hand, for a standard in situ subtalar arthrodesis, even with compression of the subtalar joint through a partially threaded lag screw, some cases may not achieve full contact between calcaneus and talus, particularly in cases with a depressed calcaneal posterior facet (Fig. 5). In our clinical study, use of fully threaded screws was 5.90 times (OR = 5.90, 95% CI = 1.42–24.49, *p* = 0.02) more likely to achieve successful bone union compared to the use of partially threaded screws. However, future prospective randomized controlled study comparing the use of fully threaded screws versus partially threaded screws placed in the same configuration is required to confirm the finding.

Biomechanical and cadaveric studies show higher compressive force, torsional stiffness, and joint rotation resistance achieved through double-screw fixation compared to a single screw [13, 14]. However, in clinical studies, no significant difference in rates of bone union between single and double screw fusion techniques has yet to be demonstrated [11, 32]. In a study of 113 patients who underwent isolated subtalar arthrodesis, the fusion rate was not significantly different between use of a single screw and double screw techniques (85.4% vs 75%, *p* = 0.37) [18]. However, only 25 (22.1%) cases were for the treatment of posttraumatic subtalar arthritis. In our study, restricted to the analysis of subtalar arthrodesis used in the treatment of posttraumatic arthritis, use of two parallel screws or the two divergent screws were 3.71 times (OR = 3.71, 95% CI = 1.05–13.14, *p* = 0.04) and 4.65 times (OR = 4.65, 95% CI = 1.23–17.53, *p* = 0.02) more likely to achieve successful bone union compared to the use of a single screw.

Biologic factors also play an important role in osseous consolidation [33]. The bony calcaneus is rich in vascular supply such that avascular necrosis of the calcaneus is extremely rare [34]. However, following open reduction of a calcaneal fracture, there is the possibility for a decrease in blood supply to the posterior facet fragment; because the collapsed posterior facet fragment is elevated and occasionally detached from the body of the calcaneus during fracture reduction and reduced into the medial fragment, creating empty space inferior to the posterior facet fragment where bone graft is known to be not necessary [35, 36]. Although this empty space can later be filled with bone, blood supply to the posterior facet fragment can be decreased and result in avascular necrosis of bone [2]. Easley et al. [2] found more than 2 mm of avascular subchondral bone at the subtalar joint in 45 (41%) out of 109 posttraumatic subtalar arthritis cases following calcaneal fracture. They identified a 38% rate of nonunion in feet with evidence of devascularized bone. In their study, every one of 30 patients with nonunion had more than 2 mm of avascular subchondral bone. Insufficient blood supply or avascular sclerotic bone at the joint can negatively affect joint fusion. Furthermore, talus is known to be low in blood vessel network and to lack collateral blood supply. Bone grafting can help to achieve early successful bone union after subtalar arthrodesis in cases of posttraumatic arthritis. Autografting may represent the better choice where there is avascular bone at the subtalar joint. In our study of posttraumatic subtalar arthritis after calcaneal fracture, 53.1% of patients with autograft achieved successful bone union within six postoperative months compared to 37.9% of those without bone grafting. Use of cancellous autograft or structural autograft was 4.72 times (OR = 4.72, 95% CI = 1.17–19.06, *p* = 0.03) and 7.12 times (OR = 7.12, 95% CI = 1.46–34.68, *p* = 0.02) more likely to achieve successful bone union compared to no graft use. In seven cases, necrotic bone at the joint was removed and resulted in a gap of more than 1 cm. In those cases, autoiliac tricortical bone was inserted and, in each case, achieved successful bone union (Fig. 3). Although it is likely that use of autograft remains superior to the use of allograft or bone substitute for achieving bone union at the graft site, morbidity at the donor site or the time required to harvest the graft may suggest reasons for opting against use of autograft [7]. Similarly, although using an allograft or bone substitute or even opting to forego grafting altogether, can give similar rates of bone union as that of autografting, when the application of an autograft can help achieve early and definite bone union, may present another indication for its use [37]. However, a future prospective randomized trial is required to warrant this finding.

The strength of this study is the large sample size of subtalar arthrodesis specifically for the treatment of posttraumatic subtalar arthritis following calcaneal fracture. Previous studies have focused on the risk factors for nonunion after subtalar arthrodesis [9]. However, to the best of our knowledge, no study yet has focused on the factors that can increase rates of fusion for cases of posttraumatic subtalar arthritis only. Moreover, our study involved multiple centers and multiple surgeons. The participation of multiple surgeons likely improves the generalizability of our findings, increasing the external validity and applicability of our results to clinical practice. However, although all participating surgeons at different centers had followed generalized principles for subtalar arthrodesis technique, there remains the possibility for a difference in outcomes due to difference in both device use and surgical technique. Surgeons themselves changed their techniques during the time to seek for better outcomes. There was not a uniform criterion for bone grafting. The use of bone grafting or numbers and types of screw depended on surgeons’ preference rather than the cases. This could have led to selection bias. With a wide variety of surgical methods, decision-making process, screw choice, and postoperative care, it may not be appropriate to test a hypothesis. The best scientific method may be a prospective randomized controlled trial. However, we believe a case series, such as this study, also has its’ value to generate a hypothesis that subsequently can be tested in studies of greater methodological rigor. We considered a range of possible factors that could affect the surgical outcome, such as the number and the types of screws used, different screw configurations, and the use of bone grafting in the multivariate logistic regression analysis. Our study did not analyze for different screw make nor diameters of screws used, which can be potential confounders in the analysis. In a biomechanical study on subtalar arthrodesis, however, increasing the screw diameter from 6.5 to 8.0 mm resulted in no additional stability of the arthrodesis [21].

CT scanning was not routinely applied to evaluate extent of bone union in this study. In an ideal comparison, bone union would be confirmed by CT scan in all cases, although this would expose many patients to unnecessary radiation. CT scans were reserved for patients with persistent pain or those without evidence of osseous consolidation on plain radiograph, as proposed by Davies et al. [38] Another limitation of the study is that we excluded patients with infection which is considered an important risk factor for nonunion. In the study, we focused more on the factors influencing successful bone union, especially those that can be controlled by surgeons. Moreover, it was not possible in this retrospective study to account for all potential confounders due to the limitations in the application of retrospective data. A future prospective randomized study with assessment of functional outcomes may confirm our findings to further guide clinical practice.

## Conclusion

Use of fully threaded screws, autograft, and two screws compared to a single screw was the factors associated with successful bone union within six postoperative months after subtalar arthrodesis for the posttraumatic arthritis. A future prospective randomized study with assessment of functional outcomes may confirm our findings to further guide clinical practice.

## Supplementary Information

**Additional file 1:** Case demographics and union rates with respect to use of partially threaded or fully threaded screws.

**Additional file 2:** Case demographics and union rates with respect to use of different screw configurations.

**Additional file 3:** Case demographics and union rates with respect to use of no graft, allograft or bone substitute, and autograft.

**Additional file 4:** Nonunion rates according to variable factors.

## Data Availability

The datasets used and/or analyzed during the current study are available from the corresponding author on reasonable request.
