# Supplementary material for: Factors influencing successful bone union of isolated subtalar arthrodesis for posttraumatic subtalar arthritis: a multicenter case series
Source: J Orthop Surg Res. 2023 Aug 2;18:559. doi: 10.1186/s13018-023-04040-9 (PMC10398992; doi:10.1186/s13018-023-04040-9)
Supplement: Supplementary file 1 — Additional file 1: Case demographics and union rates with respect to use of partially threaded or fully threaded screws. [file 13018_2023_4040_MOESM1_ESM.docx]

**Supplementary file 1**. Case demographics and unionrates with respect to use of partially threaded or fully threaded screws

|  | **Partially threaded screws**  **N=78^a^** | **Fully threaded screws**  **N=36^a^** | ***P*-value** |
| --- | --- | --- | --- |
| **Age (y)** | 50.8 ± 12.3 | 51.0 ± 10.3 | 0.94 |
| **Sex (male)** | 54 (69.2%) | 29 (80.6%) | 0.28 |
| **BMI (kg/m^2^)** | 24.1 ± 3.7 | 25.2 ± 3.3 | 0.23 |
| **Cigarette smoking** | 13 (16.7%) | 12(33.3%) | 0.05 |
| **Diabetes mellitus** | 13 (16.7%) | 3 (8.3%) | 0.23 |
| **Revision subtalar arthrodesis** | 4 (5.1%) | 4 (11.1%) | 0.25 |
| **Type of screw configuration** |  |  | <0.001 |
| **Single (1 screw)** | 28 (35.9%) | 1 (2.8%) |  |
| **Parallel (2 screws)** | 23 (29.5%) | 10 (27.8%) |  |
| **Divergent (2 screws)** | 27 (34.6%) | 25 (69.4%) |  |
| **Use of graft** |  |  | 0.004 |
| **No graft** | 24 (30.8%) | 5 (13.9%) |  |
| **Allograft, bone substitute** | 26 (33.3%) | 6 (16.7%) |  |
| **Autograft** | 28 (52.8%) | 25 (47.2%) |  |
| **Successful bony union** | 39 (50.0%) | 33 (91.7%) | <0.001 |

*^a^Values are given as the number of cases with percentages in parenthesis. Exceptions were age and BMI given as the mean ± standard deviation. Successful boneunion was defined as resolution of hindfoot pain with the presence of osseous trabecular bridging of more than 50% of the posterior facet surface without evidence for screw loosening before the six-month postoperative stage. BMI=body mass index*
