# Supplementary material for: Factors influencing successful bone union of isolated subtalar arthrodesis for posttraumatic subtalar arthritis: a multicenter case series
Source: J Orthop Surg Res. 2023 Aug 2;18:559. doi: 10.1186/s13018-023-04040-9 (PMC10398992; doi:10.1186/s13018-023-04040-9)
Supplement: Supplementary file 2 — Additional file 2: Case demographics and union rates with respect to use of different screw configurations. [file 13018_2023_4040_MOESM2_ESM.docx]

**Supplementary file 2**. Case demographics and union rateswith respect to use of different screwconfigurations

|  | **Single (1 screw)**  **N=29^a^** | **Parallel (2 screws)**  **N=33^a^** | **Divergent (2 screws)**  **N=52^a^** | ***P*-value** |
| --- | --- | --- | --- | --- |
| **Age (y)** | 46.2 ± 12.3 | 55.0 ± 10.1 | 50.9 ± 11.5 | 0.01 |
| **Sex (male)** | 19 (65.5%) | 26 (78.8%) | 38 (73.1%) | 0.50 |
| **BMI (kg/m^2^)** | 24.6 ± 2.6 | 25.1 ± 2.8 | 23.9 ± 4.5 | 0.34 |
| **Cigarette smoking** | 5 (17.2 %) | 10 (30.3%) | 10 (19.2%) | 0.38 |
| **Diabetes mellitus** | 3 (10.3 %) | 2 (6.1%) | 11 (21.2%) | 0.12 |
| **Revision subtalar arthrodesis** | 0 (0 %) | 3 (9.1%) | 5 (9.6%) | 0.23 |
| **Type of screw** |  |  |  | <0.001 |
| **Partially threaded screw** | 28 (96.6%) | 23 (69.7%) | 27 (51.9%) |  |
| **Fully threaded screw** | 1 (3.4%) | 10 (30.3%) | 25 (48.1%) |  |
| **Use of graft** |  |  |  | 0.10 |
| **No graft** | 10 (34.5%) | 8 (24.2%) | 11 (21.2%) |  |
| **Allograft, bone substitute** | 10 (34.5%) | 12 (36.4%) | 10 (19.2%) |  |
| **Autograft** | 9 (31.0%) | 13 (39.4%) | 31 (59.6%) |  |
| **Successful bony union** | 10 (34.5%) | 23 (69.7%) | 39 (75.0%) | 0.001 |

*^a^Values are given as the number of cases with percentages in parenthesis. Exceptions were age and BMI given as the mean ± standard deviation. BMI=body mass index*
