# Supplementary material for: Factors influencing successful bone union of isolated subtalar arthrodesis for posttraumatic subtalar arthritis: a multicenter case series
Source: J Orthop Surg Res. 2023 Aug 2;18:559. doi: 10.1186/s13018-023-04040-9 (PMC10398992; doi:10.1186/s13018-023-04040-9)
Supplement: Supplementary file 3 — Additional file 3: Case demographics and union rates with respect to use of no graft, allograft or bone substitute, and autograft. [file 13018_2023_4040_MOESM3_ESM.docx]

**Supplementary file 3**. Case demographics and union rateswith respect to use of no graft, allograft or bone substitute, and autograft

|  | **No bone graft**  **N=29^a^** | **Allograft or**  **bone substitutes**  **N=32^a^** | **Autograft**  **N=53^a^** | ***P*-value** |
| --- | --- | --- | --- | --- |
| **Age (y)** | 48.7 ± 13.9 | 52.3 ± 11.3 | 51.2 ± 10.5 | 0.48 |
| **Sex (male)** | 23 (79.3%) | 20 (62.58%) | 40 (75.5%) | 0.28 |
| **BMI (kg/m^2^)** | 25.4 ± 3.0 | 23.2 ± 4.5 | 24.6 ± 3.2 | 0.06 |
| **Cigarette smoking** | 8 (27.6%) | 6 (18.8%) | 11 (20.8%) | 0.68 |
| **Diabetes mellitus** | 5 (17.2%) | 6 (18.8 %) | 5 (9.4%) | 0.41 |
| **Revision subtalar arthrodesis** | 1 (3.4%) | 0 (0 %) | 7 (13.2%) | 0.05 |
| **Type of screw** |  |  |  | 0.004 |
| **Partially threaded screw** | 24 (82.8%) | 26 (81.2%) | 28 (52.8%) |  |
| **Fully threaded screw** | 5 (17.2%) | 6 (18.8%) | 25 (47.2%) |  |
| **Type of configuration** |  |  |  | 0.10 |
| **Single (1 screw)** | 10 (34.5%) | 10 (31.3%) | 9 (17.0%) |  |
| **Parallel (2 screws)** | 8 (27.6%) | 12 (37.5%) | 13 (24.5%) |  |
| **Divergent (2 screws)** | 11 (37.9%) | 10 (31.3%) | 31 (58.5%) |  |
| **Successful bony union** | 11 (37.9%) | 17 (53.1%) | 44 (61.1%) | <0.001 |

*^a^Values are given as the number of cases with percentage in parenthesis. Exceptions were age and BMI given as the mean ± standard deviation. BMI=body mass index*
