# Supplementary material for: Factors influencing successful bone union of isolated subtalar arthrodesis for posttraumatic subtalar arthritis: a multicenter case series
Source: J Orthop Surg Res. 2023 Aug 2;18:559. doi: 10.1186/s13018-023-04040-9 (PMC10398992; doi:10.1186/s13018-023-04040-9)
Supplement: Supplementary file 4 — Additional file 4: Nonunion rates according to variable factors. [file 13018_2023_4040_MOESM4_ESM.docx]

**Supplementary file 4**. Nonunion rates according to variable factors

|  | **Number of cases**  **N=114^a^** | **Nonunion rate^b^** | **Collinearity statistics** | | **Bivariate analysis: chi-square test** | | **Multivariate analysis: binary logistic regression** | |
| --- | --- | --- | --- | --- | --- | --- | --- | --- |
|  |  |  | **Tolerance** | **VIF** | **Odds ratio (95% CI)** | ***P*-value**^c^ | **Odds ratio (95% CI)** | **P-value**d |
| **Male** | 83 (72.8%) | 18 (21.7%) | 0.91 | 1.11 | 1.44 (0.48 – 4.28) | .51 |  |  |
| **Old age (>65 years)** | 12 (10.5%) | 5 (41.7%) | 0.77 | 1.29 | 3.33 (0.95 – 11.70) | .05 |  |  |
| **Overweight (BMI >25 kg/m^2)^** | 47 (41.2%) | 8 (17.0%) | 0.95 | 1.10 | 0.71 (0.27 – 1.85) | .71 |  |  |
| **Diabetes** | 16 (14.0%) | 7 (43.8%) | 0.77 | 1.31 | 3.99 (1.30 - 12.26) | .01 | 5.38 (1.51 - 19.18) | .01 |
| **Smoking** | 25 (21.9%) | 6 (24.0%) | 0.87 | 1.16 | 1.33 (0.46 – 3.86) | .59 |  |  |
| **Revision subtalar arthrodesis** | 8 (7.0%) | 1 (12.5%) | 0.92 | 1.09 | 0.55 (0.06 – 4.67) | .58 |  |  |
| **Type of screw** |  |  | 0.78 | 1.29 |  |  |  |  |
| **partially threaded screw** | 78 (68.4%) | 20 (25.6%) |  |  | Reference | - |  |  |
| **fully threaded screw** | 36 (31.6%) | 3 (8.3%) |  |  | 0.26 (0.07 – 0.95) | .03 |  |  |
| **Number of screw** |  |  | 0.83 | 1.14 |  |  |  |  |
| **Single screw** | 29 (25.4%) | 12 (41.4%) |  |  | Reference | - | Reference | - |
| **Double screws** | 85 (74.6%) | 11 (12.9%) |  |  | 0.21 (0.08 – 0.56) | .001 | 0.18 (0.06 - 0.55) | .002 |
| **Bone graft** |  |  | 0.87 | 1.20 |  |  |  |  |
| **No bone graft** | 29 (25.4%) | 11 (37.9%) |  |  | Reference | - | Reference | - |
| **Allograft or bone substitute** | 32 (28.1%) | 7 (21.9%) |  |  | 0.46 (0.15 – 1.41) | .17 | 0.40 (0.12 - 1.38) | .15 |
| **Autograft** | 53 (46.5%) | 5 (9.4%) |  |  | 0.17 (0.05 – 0.56) | .002 | 0.21 (0.06 - 0.76) | .02 |

*^a^Values are given as the number of cases with percentages in parenthesis. ^b^Nonunion was defined as the lack of bridging callus or trabeculation and continued pain for more than one year postoperatively, or any case requiring revision arthrodesis.Values are given as the number of cases with nonunion and nonunion rates in parenthesis.* ^c^*Chi-square test or Fishers’ exact test was used to determine the association between the variable factors and the nonunion.* ^d^*Multivariate logistic regression analysis was performed to determine the association between the variable factors and the nonunion. VIF=variance influence factor, CI=confidence interval, Ref=reference.*
